# EPySeg: a coding-free solution for automated segmentation of epithelia using deep learning

**DOI:** 10.1242/dev.194589

**Published:** 2020-12-23

**Authors:** Benoit Aigouy, Claudio Cortes, Shanda Liu, Benjamin Prud'Homme

**Affiliations:** 1 Aix Marseille University, CNRS, IBDM, 13288 Marseille, France; 2 Max Planck Institute for Plant Breeding Research, 50829 Köln, Germany

**Keywords:** Computer vision, Deep learning, Epithelia, Quantitative biology, Segmentation, Software

## Abstract

Epithelia are dynamic tissues that self-remodel during their development. During morphogenesis, the tissue-scale organization of epithelia is obtained through a sum of individual contributions of the cells constituting the tissue. Therefore, understanding any morphogenetic event first requires a thorough segmentation of its constituent cells. This task, however, usually involves extensive manual correction, even with semi-automated tools. Here, we present EPySeg, an open-source, coding-free software that uses deep learning to segment membrane-stained epithelial tissues automatically and very efficiently. EPySeg, which comes with a straightforward graphical user interface, can be used as a Python package on a local computer, or on the cloud via Google Colab for users not equipped with deep-learning compatible hardware. By substantially reducing human input in image segmentation, EPySeg accelerates and improves the characterization of epithelial tissues for all developmental biologists.

## Abstract (precis)

**Summary:** EPySeg is easy-to-use open-source software that applies deep-learning technology for segmentation of membrane-labelled epithelial tissues, either locally or in the cloud.

## INTRODUCTION

Epithelia are dynamic tissues undergoing dramatic shape changes throughout their development. A prerequisite for understanding these morphogenetic events is the thorough segmentation of cells constituting the tissue. To this aim, numerous semi-automated methods have been developed (Aigouy et al., 2016; Farrell et al., 2017; Cilla et al., 2015; Heller et al., 2016), but they require time-consuming manual correction to achieve optimal segmentation.

Over the past few years, deep learning, and more particularly convolutional neural networks (CNNs), has reshaped the computer vision field. In particular, deep-learning approaches should be beneficial for image segmentation because they could, in theory, reduce or even eliminate the need for end-user correction of the segmentation output. The advent of simple programming frameworks, such as Keras (https://github.com/fchollet/keras) and TensorFlow (Abadi et al., 2016 preprint), has made deep learning accessible to most developers but still excludes people lacking coding skills, preventing deep learning from being broadly adopted by the scientific community. A few attempts to bring CNNs to well-known image processing frameworks such as ImageJ or FIJI exist (Schmidt et al.; Weigert et al., 2018; Gómez-de-Mariscal et al., 2019 preprint; Schindelin et al., 2012; Schneider et al., 2012), but they require an up-to-date and adequately configured computer. More importantly, most often those powerful, yet very poorly generalizable, CNNs need to be trained *de novo* on user-provided data to work efficiently. Unfortunately, in most cases, such training cannot be done directly in FIJI or ImageJ and requires coding expertise. So far, little effort has been made to facilitate CNN training and use by regular users (von Chamier et al., 2020 preprint; Buchholz et al., 2020 preprint).

To address all these limitations, we present EPySeg, a coding-free solution to efficiently segment raw images of epithelial tissues, using a pre-trained neural network. Furthermore, EPySeg comes with a complete and straightforward graphical user interface (GUI), allowing users that are curious about deep learning, as well as more advanced users, to build and train custom networks to achieve any segmentation paradigm of interest. EPySeg is available at https://github.com/baigouy/EPySeg, and a minimal version can also be used on Google Colab (https://github.com/baigouy/notebooks) for users equipped with low-end graphics cards.

## RESULTS AND DISCUSSION

**Fig. 1.**
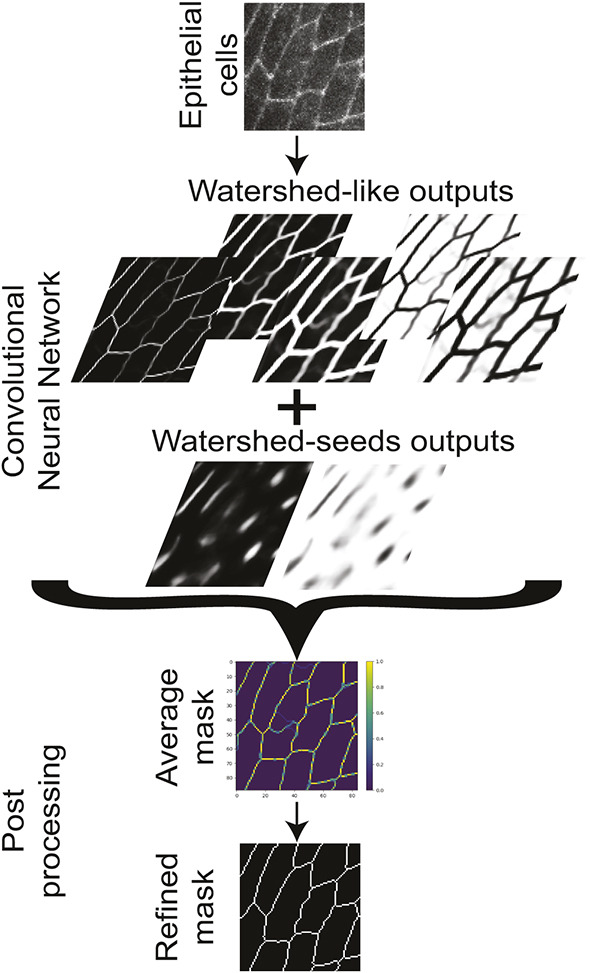
**EPySeg segmentation pipeline.** An unseen image of cells labelled with a membrane marker is provided to the EPySeg pre-trained neural network. EPySeg produces seven outputs from it: five of them are watershed-like outputs, while the remaining two are watershed seeds. Those seven outputs are used to generate seven watershed masks. Upon thresholding the average of these seven masks, we obtain a refined mask.

In this study, we set out to develop a software that uses deep learning to automate the time-consuming segmentation of 2D epithelial tissue images. We selected LinkNet architectures, because they are known to perform well at image segmentation tasks (Chaurasia and Culurciello, 2017; also see Materials and Methods). Our network was trained on a large number of images of very divergent fly epithelia acquired using several microscopy setups (see Materials and Methods) to allow our segmentation paradigm to be robust and able to segment a broad range of epithelial tissues. Cell segmentation was generated using the watershed algorithm (Vincent and Soille, 1991), followed by careful manual curation to remove errors (see Materials and Methods). In EPySeg, this watershed segmentation was converted into a set of five watershed-like segmentations and two watershed seeds (see Materials and Methods) that the EPySeg neural network is trained to generate when given an input epithelial image (Fig. 1). The seven outputs generated by the neural network are combined into a single watershed mask upon averaging and thresholding (Fig. 1). This mask then corresponds to an optimized watershed-like segmentation of the tissue.

**Fig. 2.**
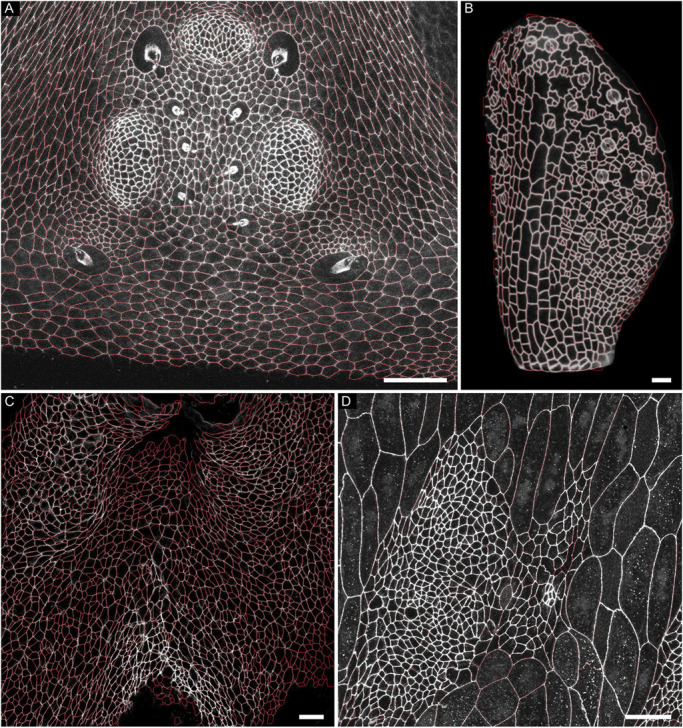
**EPySeg segmentation of unseen epithelium images.** (A-D) EPySeg segmentation (red) overlaid on unseen images. (A) Segmentation of the *Drosophila* head epithelium, including ocelli, labelled with E-cadherin:GFP (greyscale). (B) Segmentation of the fourth leaf of a plasma membrane-labelled *Arabidopsis thaliana* plant at 7 days post germination (greyscale, UBQ10::acyl:tdTomato). (C) Segmentation of Phalloidin-labelled (greyscale) vertebrate dorsal pericardial wall epithelium (Cortes et al., 2018; Francou et al., 2017). (D) Segmentation of the *Drosophila* abdominal region surrounding a histoblast nest, labelled with E-cadherin:GFP (greyscale). Scale bars: 25 µm.

EPySeg, although trained exclusively on fly epithelia, can efficiently segment evolutionarily distant 2D epithelial tissues imaged with different optics (Fig. 2; Table S1). We compared our software to Cellpose, the only software available to date that can segment cells without the need for prior model training (Stringer et al., 2020 preprint). On average, EPySeg outperformed Cellpose on epithelia in two ways: its approximation of the cell outline was more precise than that of Cellpose (Fig. S1, Table S1), and it missed fewer cells (Fig. 2; Fig. S2; Table S1). We note, however, that unlike Cellpose, EPySeg was not able to segment cells in culture (Table S1) and is likely to be less efficient at segmenting non-cellular objects than Cellpose, because it was not trained to accomplish such tasks.

Finally, to make our epithelial segmentation tool easily accessible to a broad audience, we created a GUI and detailed documentation for its use (https://github.com/baigouy/EPySeg). This interface allows for building, training and running CNNs. It is built in such a way that non-expert users can rely on the default settings to easily train a network and gain knowledge on using deep learning for image analysis, whereas advanced users can visually fine-tune parameters to achieve optimal results. Because the majority of computers available in research labs are not deep learning-ready, we also provide a minimal user interface to run EPySeg online, in Google Colab, granting a broader audience access to deep-learning approaches (https://github.com/baigouy/notebooks).

## MATERIALS AND METHODS

### Recommended equipment

The EPySeg CNN was trained on a Dell Precision 7820 with 64 GB RAM, equipped with a Nvidia GeForce RTX 2070 graphic card with 8 GB RAM. Most training lasted less than 12 h. We could also successfully train and run our CNN on Google Colab, hereby providing a good alternative for users with deep learning-incompatible systems.

### Data

The EPySeg CNN was trained on several *Drosophila* epithelia stained with E-cadherin:GFP that largely diverged from one another. One training set consisted of tissue from embryonic stages, where E-cadherin staining in epithelia appeared dotted (Tepass and Hartenstein, 1994; Truong Quang et al., 2013; Cavey et al., 2008) and the boundary-to-cytoplasm signal ratio was low. Another training set used pupal wing tissue, where E-cadherin staining appeared continuous and presented a higher boundary-to-cytoplasm ratio, except for stretched cells. Finally, our third training set contained images of the fly abdomen, including giant, polyploid, larval cells and tiny histoblast nest cells (Madhavan and Madhavan, 1980), in order to have a network that can segment cells without a size bias. Input images were either max- or stack-focuser projections (using Stack Focuser ImageJ plugin; https://imagej.nih.gov/ij/plugins/stack-focuser.html) of all or part of confocal *z*-stacks of epithelial tissues. Segmented cell outlines, serving as ground truth for training the network and for evaluating the segmentation quality, were generated using the watershed algorithm of Tissue Analyzer (Aigouy et al., 2016; Vincent and Soille, 1991). Importantly, we paid a lot of attention to the quality of the segmentation masks fed to the CNN, and we cropped out regions where segmentation quality was poor as well as regions that were not segmented (e.g. cells adjacent to the tissue of interest) in order not to perturb the learning process. For training the model, every watershed segmentation mask was used to generate seven images, the first image was the curated watershed mask itself, the second and third were the same watershed mask after one or two binary dilations, respectively. The fourth and fifth images were the negatives of the second and third images, respectively (akin to a non-cellular background). The sixth image was generated to contain a single seed (group of pixels) per cell, scaled by the cell size. The seventh image was the negative of the sixth image. The model was asked to reproduce these seven outputs for any given input. Two of the three training datasets were acquired on regular Leica or Zeiss confocal microscopes (Leica SP2 and LSM 510, respectively), whereas the third dataset was acquired on a spinning-disc microscope (Roper) to expand the breadth of optics used. The plant sample used for testing our segmentation is the fourth leaf of a 7 days after germination transgenic *Arabidopsis thaliana* labeled with UBQ10::acyl:tdTomato (modified from the construct by Willis et al., 2016). The vertebrate test sample is a ventral view of the dorsal pericardial wall epithelium stained with Phalloidin. Both test samples were acquired using a Leica SP8 upright confocal microscope and a Zeiss LSM 780, respectively. The fly wing and abdominal test samples were acquired using an Olympus FV-1000 confocal microscope. The fly head sample was acquired using an LSM 510 microscope.

### Data augmentation

To further increase the size of our training set for deep learning (images and cells) and to prevent the neural network from overfitting, we used data augmentation: we randomly applied the same transformation (rotation, translation, magnification, flip, …) within a given range to both the input and output images. Our data augmentation algorithm currently supports 2D and 3D images (only 2D images were used in this study).

### CNN building and training

Our CNN was generated using the segmentation_models library from (https://github.com/qubvel/segmentation_models) and relies on TensorFlow and Keras. We used a LinkNet (Chaurasia and Culurciello, 2017) architecture with a VGG16 encoder (Simonyan and Zisserman, 2015, preprint). We found that this encoder, known to perform well at classification tasks, was also very efficient at segmenting epithelia. Of note, the detailed model architecture is available in the log window of the software upon loading the model. The network was trained for 300 epochs on the complete training set at every epoch. We used Adam (Kingma and Ba, 2017 preprint) as the optimizer with an initial 10^−3^ learning rate for the first 150 epochs and a 10^−4^ learning rate for the next 150 epochs. The network was trained with a batch size of 24 images and a tile size of 256 pixels in width and height. We chose the intersection over union (IoU), also called the Jaccard index, for the loss function, because it is particularly well suited to evaluate differences between binary images (Rahman and Wang, 2016).

### Segmentation quantification

To measure the accuracy of cell segmentation (i.e. quality of the cell mask) we used the SEG score (Ulman et al., 2017). Briefly, this measure evaluates the average amount of overlap between the reference segmentation and the corresponding neural network-generated segmentation. As a measure for segmentation quality (i.e. an evaluation of over- and under-segmentation), we used the average precision score (*AP*) defined as *AP*=*TP*/(*TP*+*FP*+*FN*), where *FP* corresponds to over-segmented cells and *FN* correspond to under-segmented cells. *TP*, the properly segmented cells, are defined as segmented cells having an IoU score ≥0.7 when compared with the corresponding ground truth cell.

### Software

The software was entirely coded in Python 3. The graphical user interface was made with PyQT5 (Riverbank). The source code of our tool along with installation instructions can be found at https://github.com/baigouy/EPySeg.

### Ethical approval

Animal experiments were carried out in agreement with national and European laws and approved by the Ethics Committee for Animal Experimentation of Marseille and the French Ministry for National Education, Higher Education and Research.

## Supplementary Material

Supplementary information

Reviewer comments
