# Supplementary material for: EPySeg: a coding-free solution for automated segmentation of epithelia using deep learning
Source: Development. 2020 Dec 23;147(24):dev194589. doi: 10.1242/dev.194589 (PMC7774881; doi:10.1242/dev.194589)
Supplement: Supplementary information [file develop-147-194589-s1.pdf]

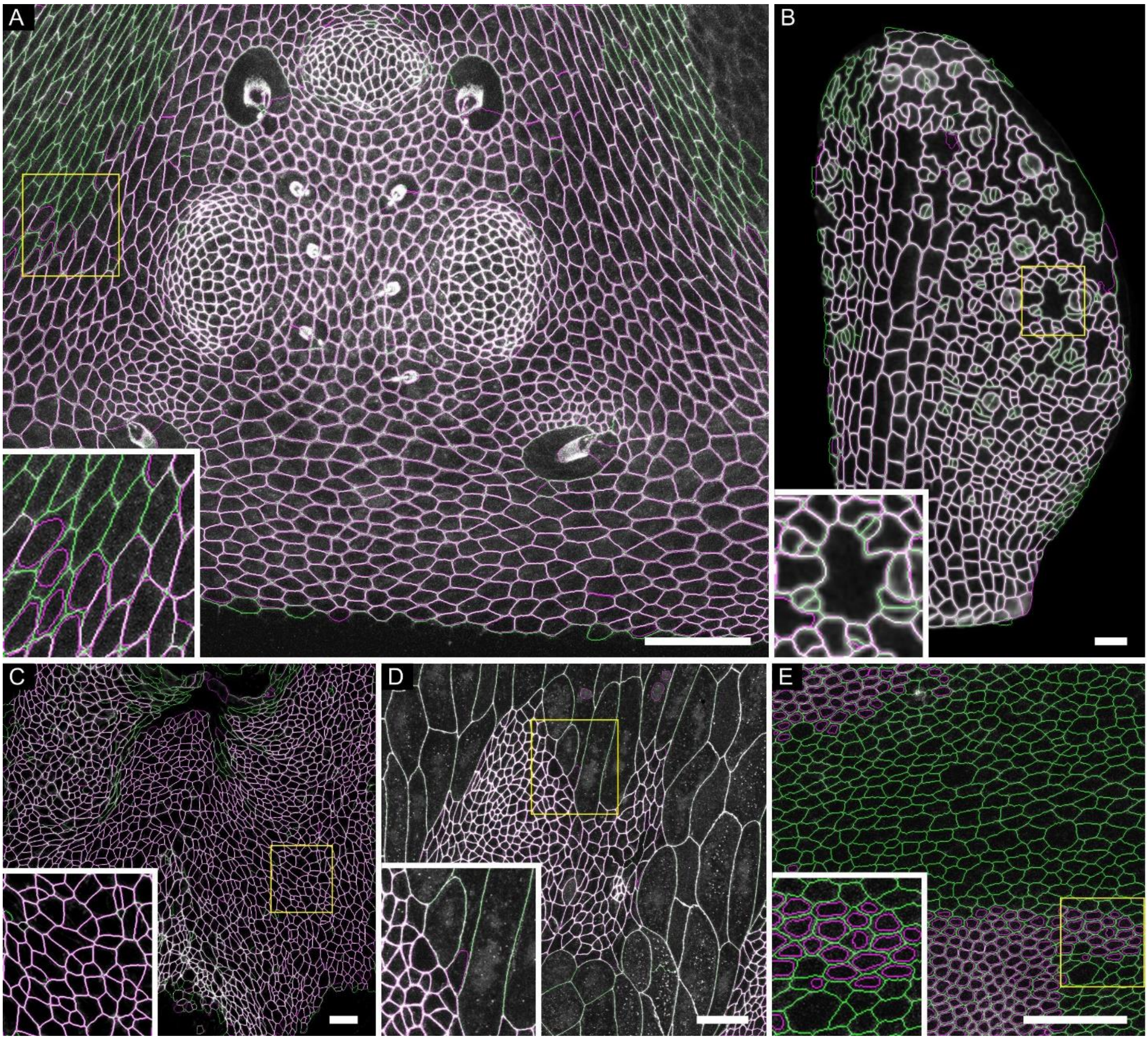

Figure S1: EPySeg segmentation of cell outlines on various epithelia

Comparison of cell outlines generated by EPySeg (green) and Cellpose (magenta) overlaid over original images. **(A)** E-cadherin:GFP staining of the fly head epithelium including fly ocelli. **(B)** Image of the fourth leaf of a seven days after germination plant. **(C)** Vertebrate epithelial cells. The image is a ventral view of the apical surface of splanchnic mesodermal cardiac progenitor cells in the dorsal pericardial wall of a mouse embryo at embryonic day 9.5, after removal of the heart tube. **(D)** Fly abdomen showing a histoblast nest surrounded by giant larval cells. **(E)** Image of the central region of drosophila wing epithelium. Insets show close-up views of the segmentations. Of note, EPySeg approximation of the cell outline is more precise than that of Cellpose, see inset in **(E)**. There, Cellpose detects the cell cytoplasm or a part of it rather than the cell outline, unlike EPySeg, resulting in a significant shift of the mask (magenta) with respect to the membrane maximum intensity. Altogether EPySeg detects more cells than Cellpose. Scale bars represent 25 µm.

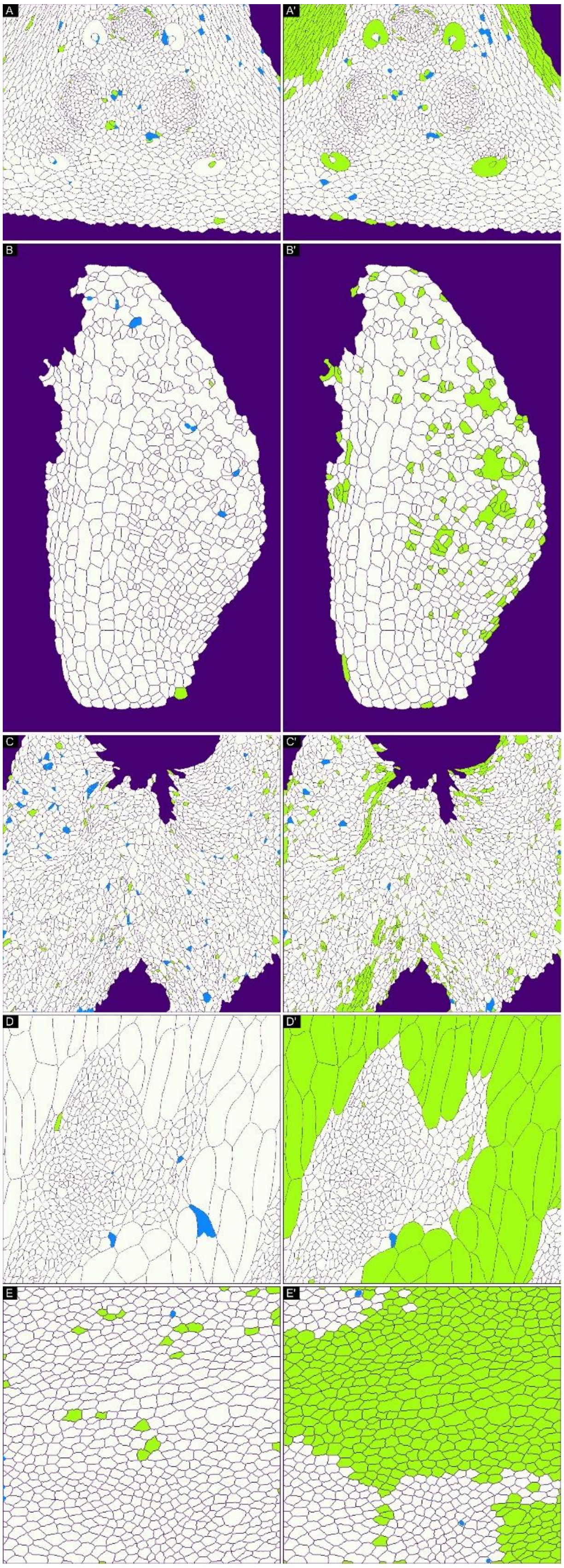

Figure S2: A comparison of EPySeg and Cellpose segmentation

Development • Supplementary information

Side by side comparison of EPySeg (left panels) and Cellpose (right panels) over- (blue) and under-segmentation (green) overlaid over the ground truth segmentation (see also **Table S1** for average precision quantification). Images in **(A, A')**, **(B, B')**, **(C, C')**, **(D, D')**, **(E, E')** correspond to images **(A, B, C, D, E)** in **Fig. S1**, respectively. **(A, A')** Cellpose did not segment stretched cells of the fly head. **(B, B')** Cellpose fails to segment non-convex (complex) and small cells in the plant epithelium. **(C, C')** Cellpose does not segment most small cells in the vertebrate epithelium. **(D, D')** Cellpose segmentation eludes all the giant larval cells from the fly abdomen. **(E, E')** Cellpose misses dim cells in the central region of the drosophila wing. Altogether, both tools show little over-segmentation (blue).

Table S1. Quantitative comparison of the segmentation efficiency of EPySeg and Cellpose on cells and tissues. The AP score is a measure of the efficiency of detection of cells by the algorithms. The AP score, that ranges between 0 and 1, is computed with an IoU of 0,7. the SEG score, that also ranges between 0 and 1, is a measure of the quality of the segmentation mask as compared to the ground truth. EPySeg scores were computed using default software settings. Cellpose scores were computed after the optimal cell size was determined by Cellpose. A representative image from the test sets labelled with asterisks is shown in Fig. 2 and Fig. S1.

Click here to Download Table S1

Development • Supplementary information
