# Supplementary material for: EPySeg: a coding-free solution for automated segmentation of epithelia using deep learning
Source: Development. 2020 Dec 23;147(24):dev194589. doi: 10.1242/dev.194589 (PMC7774881; doi:10.1242/dev.194589)
Supplement: Reviewer comments [file develop-147-194589_review_history.pdf]

EPySeg: a coding-free solution for automated segmentation of epithelia using deep learning

Benoit Aigouy, Claudio Cortes, Shanda Liu and Benjamin Prud'homme
DOI: 10.1242/dev.194589

Editor: James Briscoe

Review timeline
Original submission: 30 June 2020
Editorial decision: 14 August 2020
First revision received: 23 October 2020
Accepted: 17 November 2020

Original submission

First decision letter

MS ID#: DEVELOP/2020/194589

MS TITLE: EPySeg: a coding-free solution for automated segmentation of epithelia using deep learning

AUTHORS: Benoit Aigouy and Benjamin Prud'homme

I have now received all the referees' reports on the above manuscript, and have reached a decision. The referees' comments are appended below, or you can access them online: please go to BenchPress and click on the 'Manuscripts with Decisions' queue in the Author Area.

As you will see, the referees recognise the potential utility of your software, but have some significant criticisms and recommend a substantial revision of your manuscript before we can consider publication. All three referees make several suggestions to improve the documentation of the software. These include requests to explain various parameters and details of the methods. In addition, the software needs to be demonstrated on different datasets, particularly non-Drosophila data, to show its limitations and provide a fairer comparison with Cellpose. This will be crucial for readers so that they can assess the likely usefulness of the software to their systems.

If you are able to revise the manuscript along the lines suggested, which will involve further analyses, I will be happy receive a revised version of the manuscript. Your revised paper will be re-reviewed by one or more of the original referees, and acceptance of your manuscript will depend on your addressing satisfactorily the reviewers' major concerns. Please also note that Development will normally permit only one round of major revision.

We are aware that you may currently be unable to access the lab to undertake experimental revisions. If it would be helpful, we encourage you to contact us to discuss your revision in greater detail. Please send us a point-by-point response indicating where you are able to address concerns raised (either experimentally or by changes to the text) and where you will not be able to do so within the normal timeframe of a revision. We will then provide further guidance. Please also note that we are happy to extend revision timeframes as necessary.

Please attend to all of the reviewers' comments and ensure that you clearly highlight all changes made in the revised manuscript. Please avoid using 'Tracked changes' in Word files as these are lost

© 2020. Published by The Company of Biologists under the terms of the Creative Commons Attribution License (https://creativecommons.org/licenses/by/4.0/).

in PDF conversion. I should be grateful if you would also provide a point-by-point response detailing how you have dealt with the points raised by the reviewers in the 'Response to Reviewers' box. If you do not agree with any of their criticisms or suggestions please explain clearly why this is so.

Reviewer 1

Advance summary and potential significance to field

In the present manuscript, the authors offer a simple and accessible software tool to allow users with no programming experience to access deep learning methodologies to segment imaging data from ImageJ files, which can be generated from any imaging platform. The democratisation of AI image segmentation is a worthy and timely goal, as these tools are in the process of revolutionising data analysis in a number of fields, yet the difficulty of implementing these approaches without access to skilled programmers and GPU clusters are likely to put off many potential users. The authors propose an online version through Google Colab, as well as a GUI that supports both using pre-trained networks and training several network architectures using self-generated datasets.

We tested EPSyG using our own epithelial movie datasets. The Google platform was easy to use and relatively fast. Compared with our own U-net tool, EPSyG performed very commendably, especially considering that it had not been trained on our dataset. Given the short time span of a review, we did not have time to extensively test the training feature. Overall, this is a very useful package that looks likely to allow many new users to try and play around with deep learning methods. I would therefore be happy to recommend publication in Development, provided the points below are addressed.

Comments for the author

1. The example provided is of a very high-quality image of the fly head. The authors should show how robust the software is with more messy/noisy datasets, using the same metrics applied to the example showed by the authors.

2. The authors should provide access to the training datasets so that users can appreciate the type of data that was used, and decide whether or not to do further training with their own data.

3. The programme includes two network architectures, but it is not clear what the differences are. The authors should explain this in the manuscript. The outputs from the two architectures are also different (eg: “predict” (which itself contains different intermediate steps for one of the models) versus “refined predict”), so the authors should explain what the different outputs correspond to for non-specialist users.

4. The authors could improve the explanations of some of the parameters that the user is asked to set when running the code (eg: step 10, normalisation methods, clip intensity…). Ideally, they would write a small blurb for each of these that the user can access by hovering over the parameter or clicking on a question mark link next to the parameter.

Reviewer 2

Advance summary and potential significance to field

Aigouy and Prud'Homme present a simple tool for segmentation of cells in confocal images of Drosophila epithelia. The paper presents standard computer vision approaches and its value lies predominantly in the ease-of-use of the software. An open source Python package is provided, as well as an environment for Google Collab. The authors stress that deployment of the software is coding free while allowing for extension by qualified users.

In principle, the paper describes a useful tool for the developmental biology community. Novelty from the point of view of computer vision is lacking. The authors select published CellPose software as a benchmark. This is however not a particularly fair comparison, since CellPose is a much more

© 2020. Published by The Company of Biologists under the terms of the Creative Commons Attribution License (https://creativecommons.org/licenses/by/4.0/).

broadly applicable segmentation tool, trained on significantly more diverse data. EPySeq is trained exclusively on Drosophila E-cadherin data and with one exception (fish, presumably EVL epiboly, very simple sample to segment) applied to similar Drosophila datasets. It is expected that such networks will perform better under these circumstances. Moreover, the data presented in Figure 2 show that CellPose fails predominantly on large holes in the epithelium which is trivial to fix. This is somewhat diminishing the need to switch to the new framework.

Comments for the author

Nevertheless, the paper is fixable. I suggest to show much more of the datasets (that are currently only summarised in a supplementary Table, it should be in the main text) and to apply the framework to non-Drosophila data to show its limitations. The title and abstract should embrace the limitations of the networks to Drosophila epithelia and similar datasets.
User-friendliness of the software should be demonstrated with screen shots and a proper, tutorial style documentation in the supplements.

Minor comments:
Introduction:
- "rewamped" is a sloppy, spoken language.
- the use of CNN by no means alleviates the need for user correction of segmentation!
- it is usually not necessary to train the networks on "big data". In fact authors did not train their networks on "big data", the statement is a misleading use of a buzzword.
- authors make a confusing statement about how manual correction of watershed result SHOULD improve network training. I am not sure what they mean and if it is an important point, quantitative evidence must be presented.
- "decently trained network" is not a appropriate expression
- "we flawlessly trained and run ... " is not a scientific statement.
- the image quality of Figure 1 is low. Also, its message is trivial and should be expanded to highlight the strength of the contribution, i.e. user friendly software and GoogleCollab.
- Figure 2 should be significantly expanded, showing more results on representative datasets.
- it is not clear what offset is referred to in the inset of the panel A. I don't see any Cellpose outlines.

<u>Reviewer 3</u>

Advance summary and potential significance to field

I have difficulties identifying advances made in this paper.

Comments for the author

The submitted manuscript tackles a very important analysis step in many research projects. The presented work is in my view, unfortunately, not yet ready for publication.

Main points of concern:

- The quantification of obtained results is insufficient to understand where strength and weaknesses of the presented method lie.
- The contribution by the authors is to make an existing network and training available via an open source software package. No new technical tricks are introduced and even the combination of modules that are hooked up is very default and bears zero novelty.
- Results are only compared to results obtained by Cellpose, and I expect that the pre-trained Cellpose model from Janelia Farms was used (the manuscript did not mention to have trained Cellpose for the specific data). This is bad because the comparison is flawed. While EPySeg is allowed to train on data very similar to the validation data, Cellpose must make the best out of the very different data it was trained on. Since Cellpose is also open source, nothing stops the authors from training on the same body of data and then do a proper comparison.

© 2020. Published by The Company of Biologists under the terms of the Creative Commons Attribution License (https://creativecommons.org/licenses/by/4.0/).

- Cellpose is not the only baseline that would be interesting to compare to. In fact, Cellpose is not even the most important baseline method. Obvious baselines would e.g. be a 3-Class U-Net (very basic and known to everyone), StarDist (Weigert et. al.), or DenoiSeg (Buchholz, Prakash, et al.).
- The authors suggest to use the trained EPySeg model as a "generalist neural network" (page 1). While in the context of segmentation this can indeed work ok, it is clear that this comes with strong limitations. New data that is different to the training data will not work well at all and this is also the reason why the comparison with Cellpose is so deeply flawed.
- The description of augmentation and training is insufficient. Whats the learning rate? Does it adapt during training? What was the optimizer, what's the precise network architecture? How can IoU be the loss (IoU is non-differentiable, see also https://stackoverflow.com/questions/40475246/why-does-one-not-use-iou-for-training). All data is presented at each epoch, but does that mean all pixels are presented (since small tiles are fed)? Are the same augmented versions shown over and over, or is the augmentation different between epochs? Which dataset was using which patch and batch size? Etc. etc.
- The 'segmentation quality' metric introduced is strange and it is really not needed to introduce such a metric (there is already too many around that even make sense).
 - The metric is: (#correct - #overseg - #underseg)/#truecells. Now, each over or undersegmentation removes 1 from #correct, but causes another subtraction by 1 in the nominator. Hence, this metric will be 0 as soon as half the cells are over or undersegmentations. Weird! I suggest for example to use AP scores or any other established metric.

Other concerns:

- All work only applies to 2D data and this might go into the title to not elicit false hopes.
- Several claims in the paper are not right or overly emphasized.
 - Page 1: "Training cannot be done directly in FIJI/ImageJ" - yes, it can! DenoiSeg comes with such an option (https://imagej.net/DenoiSeg).
 - Page 1: I'm not sure if it is fair to say that the computer vision field got "revamped" at certainly the need for user correction is by far not "alleviated". Deep learning improved things, but segmentation remains a hard problem that is by far not automatically solvable.
 - "the majority of scientific computers are not deep learning-ready", depending on what the authors mean, I might have to strongly disagree. Today it is hard to even buy a laptop that is not powerful enough for a number of useful deep learning (please exclude Mac computers here, but for very different reasons). Anyway, every single microscope workstation should be more then enough for EPySeg, Cellpose, et al.
- I'm not sure about the validity of the hypothesis that EPySeg training is working better due to the non-human (but Watershed) origin of the training data. I would like to see such a claim backed up by adequate control experiments. It would, for example, be very interesting to see if a network trained on such data has the tendency to put the outline of cells at roughly constant intensity values (such as Watershedding does). Anyways, at all the crucial places the network is again trained on user annotations (curations) and the argument seems not to hold any longer.
- Authors say that EPySeg does not work on cells in culture. This is of course true, because it was not trained for this use-case. Still, could a version of EPySeg exist that was trained on this data? If so, why not do it and serve a much larger community. If not - why not?
- The caption of Figure 2 does not write their own method name correctly...
- A table to compare results would be important. The supplementary table comes without caption and must be enriched by other baselines to make sense.
- One would wish for many more qualitative examples of inputs and results in the supplement.

First revision

Author response to reviewers' comments

We would like to thank the reviewers for their useful and constructive comments on our manuscript. In the revised version, we believe we have successfully addressed the comments of all three reviewers. Mainly, we have greatly improved the documentation of our tool. Also, we now

© 2020. Published by The Company of Biologists under the terms of the Creative Commons Attribution License (https://creativecommons.org/licenses/by/4.0/).

demonstrate that the use of our tool is not restricted to segmenting fly epithelia and does not require the model to be retrained. Finally, we make two of our training datasets publicly available.

Reviewer 1 Advance Summary and Potential Significance to Field:

In the present manuscript, the authors offer a simple and accessible software tool to allow users with no programming experience to access deep learning methodologies to segment imaging data from ImageJ files, which can be generated from any imaging platform. The democratisation of AI image segmentation is a worthy and timely goal, as these tools are in the process of revolutionising data analysis in a number of fields, yet the difficulty of implementing these approaches without access to skilled programmers and GPU clusters are likely to put off many potential users. The authors propose an online version through Google Colab, as well as a GUI that supports both using pre-trained networks and training several network architectures using self-generated datasets.

We tested EPSyG using our own epithelial movie datasets. The Google platform was easy to use and relatively fast. Compared with our own U-net tool, EPSyG performed very commendably, especially considering that it had not been trained on our dataset. Given the short time span of a review, we did not have time to extensively test the training feature. Overall, this is a very useful package that looks likely to allow many new users to try and play around with deep learning methods. I would therefore be happy to recommend publication in Development, provided the points below are addressed.

Reviewer 1 Comments for the Author:

1. The example provided is of a very high-quality image of the fly head. The authors should show how robust the software is with more messy/noisy datasets, using the same metrics applied to the example showed by the authors.

In order to challenge further our tool with unseen model organisms, tissues and staining, we used a plant leaf sample labelled with a plasma membrane marker (A. thaliana UBQ10::acyl:tdTomato) and a vertebrate heart sample labelled with phalloidin (actin staining). Of note, the plant sample presents numerous irregular/wiggly cells and the vertebrate heart sample provides a complex mix of adjacent small and big cells. In addition, we show an image of the *Drosophila* histoblast nest that contains remarkable cell size differences with giant polyploid larval cells and tiny pupal epithelial nest cells. Also, we provide a difficult case of the fly wing with very dim stretched cells **(sup Fig. 1E)**. Altogether we believe these new samples (including images the model was not trained for) provide several segmentation challenges (dim, stretched, wiggly cells and tissues with a high area variability and or a combination of these features) and we are glad to see that our tool performs well on all images **(Fig. 2, sup. Fig. 1 and sup. Fig. 2)**.

2. The authors should provide access to the training datasets so that users can appreciate the type of data that was used, and decide whether or not to do further training with their own data.

In order to address the reviewer comment, we are now releasing two training datasets out of the three we used (we don't own the copyright for the remaining dataset so we cannot release it). In addition, we are also providing several of our test files so that the users can visually evaluate whether their data resembles one of the already tested ones. These additional datasets and images can be found here (https://gitlab.com/baigouy/models). More generally, we are confident that any membrane marker should be sufficient to segment cells with our tool.

3. The programme includes two network architectures, but it is not clear what the differences are. The authors should explain this in the manuscript. The outputs from the two architectures are also different (eg: "predict" (which itself contains different intermediate steps for one of the models) versus "refined predict"), so the authors should explain what the different outputs correspond to for non-specialist users.

Bearing in mind that the simpler the better, we have now reduced the number of pre-trained models in our software to one, especially because the remaining model most often outperforms the other one and is more constant on all test samples. Thereby we are now left with a single architecture: a Linknet architecture (https://arxiv.org/abs/1707.03718) with a vgg16 encoder. Please note that the

© 2020. Published by The Company of Biologists under the terms of the Creative Commons Attribution License (https://creativecommons.org/licenses/by/4.0/).

complete model architecture is printed in the log window of the software upon loading (the detailed architecture/log is given in an answer to reviewer 3, search for **#Model architecture**). Because of its length and complexity, and availability in the log window of the software, we chose to not include the model organization in the manuscript.

“Predict” and “refine” are now explained in the documentation (within the software in the ‘predict’ help window and online https://github.com/baigouy/EPySeg/blob/master/epyseg/deeplearning/docs/predict.md) and in the software help button located on the side of the refine box. Briefly, “Predict” saves the raw output of the model that consists of 5 watershed-like segmentations and 2 watershed-like seeds. Most often (but not always) this raw data is not good enough to be used directly as a segmentation mask (after thresholding) and needs be further processed in ‘refine’ to generate an optimized mask (see also **Fig. 1**).

4. The authors could improve the explanations of some of the parameters that the user is asked to set when running the code (eg: step 10, normalisation methods, clip intensity…). Ideally, they would write a small blurb for each of these that the user can access by hovering over the parameter or clicking on a question mark link next to the parameter.

We now provide a clear and complete documentation for all the parameters of the software through a careful selection of internet links along with some documentation we wrote ourselves). The help is accessible from within the software by clicking the ‘question marks/help’ buttons as suggested by the reviewer. In addition, we provide three complete tutorials, one showing how to train a model from scratch, another showing how to further train a pre-trained model and the last one showing how to use a pre- trained model for segmentation (https://github.com/baigouy/EPySeg/blob/master/epyseg/deeplearning/docs/getting_started.md, https://github.com/baigouy/EPySeg/blob/master/epyseg/deeplearning/docs/getting_started3.md, https://github.com/baigouy/EPySeg/blob/master/epyseg/deeplearning/docs/getting_started2.md)

Reviewer 2 Advance Summary and Potential Significance to Field:

Aigouy and Prud'Homme present a simple tool for segmentation of cells in confocal images of Drosophila epithelia. The paper presents standard computer vision approaches and its value lies predominantly in the ease-of-use of the software. An open source Python package is provided, as well as an environment for Google Collab. The authors stress that deployment of the software is coding free while allowing for extension by qualified users.

In principle, the paper describes a useful tool for the developmental biology community. Novelty from the point of view of computer vision is lacking. The authors select published CellPose software as a benchmark. This is however not a particularly fair comparison, since CellPose is a much more broadly applicable segmentation tool, trained on significantly more diverse data. EPySeq is trained exclusively on Drosophila E-cadherin data and with one exception (fish, presumably EVL epiboly, very simple sample to segment) applied to similar Drosophila datasets. It is expected that such networks will perform better under these circumstances.

Cellpose and StarDist, for example, are basic Unet models with varying number of model outputs and post- processing. We use a different architecture (Linknet) and have also different outputs and post-processing too, so altogether our tool is not less nor more innovative, than the afore mentioned tools, from the point of view of computer vision.

Regarding the fly specificity of our model, we now show that EPySeg, even though trained specifically on fly tissues labelled E-cadherin, does perform well on plant and vertebrate tissues labelled with very different plasma-membrane markers (see Fig. 2, sup. Fig.1 and Table 1). We also added a sentence in the main text acknowledging the versatility of the Cellpose tool: EPySeg is ‘likely to be less efficient at segmenting non-cellular objects than Cellpose’. Altogether, we hope this addresses the reviewer concerns.

Moreover, the data presented in Figure 2 show that CellPose fails predominantly on large holes in the epithelium which is trivial to fix. This is somewhat diminishing the need to switch to the new

© 2020. Published by The Company of Biologists under the terms of the Creative Commons Attribution License (https://creativecommons.org/licenses/by/4.0/).

framework.

The revised **sup. Fig.1, sup. Fig. 2 and Table 1** show that EPySeg outperforms CellPose in several cases: stretched cells, when cells in the tissue have different sizes, when the cell borders are not regular. Clearly some if not most of these segmentation errors can't be fixed even with further training of Cellpose as they are inherent features of the Cellpose method (This point is also addressed in more details later). Also, we tried tuning the cellpose 'flow_threshold ' and 'cellprob_threshold' parameters but never achieved better segmentation of large holes (we assume the Cellpose 'cell probability' must go to 0 in these regions or the size tweaks of Cellpose simply do not allow cells in these regions to be properly segmented).

Reviewer 2 Comments for the Author:

Nevertheless, the paper is fixable. I suggest to show much more of the datasets (that are currently only summarised in a supplementary Table, it should be in the main text) and to apply the framework to non- Drosophila data to show its limitations. The title and abstract should embrace the limitations of the networks to Drosophila epithelia and similar datasets. User-friendliness of the software should be demonstrated with screen shots and a proper, tutorial style documentation in the supplements.

In the revised manuscript, we have added new test images in the main **Figure 2** (and in the supplement) and make public two of our training datasets. We now show that our tool is not restricted to be used with *Drosophila* epithelia even though it was trained only with fly samples. Indeed, we now show that our model can, for example, very efficiently segment a vertebrate tissue (heart) and a plant epithelium, and should be able to segment any membrane-labelled cell. Also, as suggested by reviewer 2, we have added three classical tutorials with screenshots (also accessible from within the software 'help' tab) demonstrating how the software should be used, the first tutorial shows how one can use our model to segment epithelial cells using our pre-trained model, the second one shows how to train a model from scratch and the third one shows how one can further train our pre-trained model (https://github.com/baigouy/EPySeg/blob/master/epyseg/deeplearning/docs/getting_started2.md ,https://github.com/baigouy/EPySeg/blob/master/epyseg/deeplearning/docs/getting_started.md, https://github.com/baigouy/EPySeg/blob/master/epyseg/deeplearning/docs/getting_started3.md) .

Minor comments:

Introduction:

- "rewamped" is a sloppy, spoken language.

We replaced 'revamped' with 'reshaped'

- the use of CNN by no means alleviates the need for user correction of segmentation!

It definitely does, as an example when we compare segmentation with Tissue Analyzer (with optimized parameters) to that of EPySeg with the default settings, we can see that the number of cells to manually correct goes down from 1065 to 7 **(See figure below)**. Of note, the image contains 713 cells but the giant larval cells are highly over-segmented. Altogether, this constitutes a substantial reduction in the number of corrections the user has to do to obtain an optimal segmentation, and therefore a significant gain of time. Although, we agree with the reviewer that manual correction is still needed, especially for training deep learning, therefore we replaced 'user' by 'end-user' to distinguish the person that trains the algorithm from the one that merely uses it. We toned down the sentence to reflect that deep learning approach could, 'in theory, reduce or even eliminate end-user corrections'.

© 2020. Published by The Company of Biologists under the terms of the Creative Commons Attribution License (https://creativecommons.org/licenses/by/4.0/).

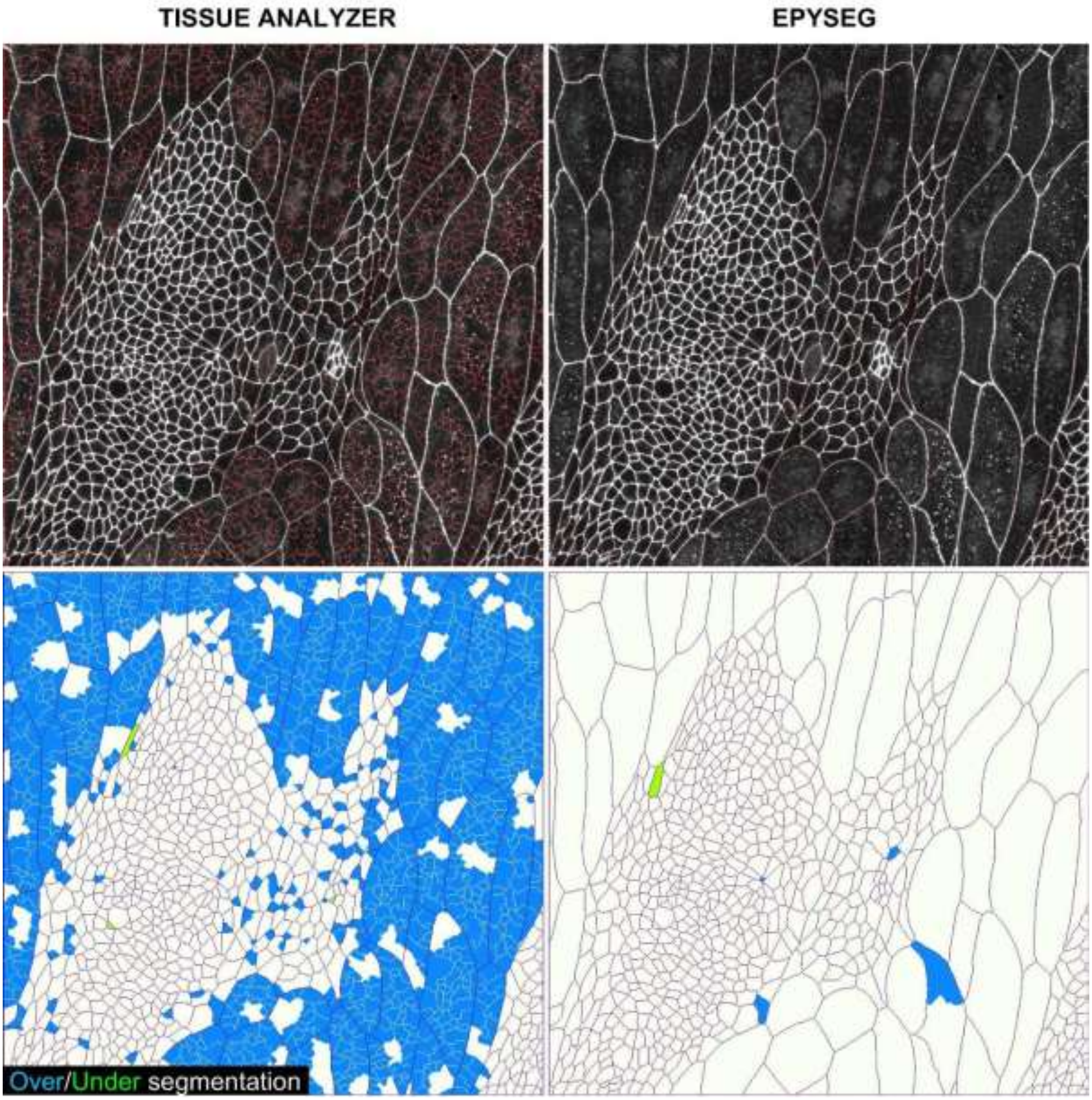

-

- it is usually not necessary to train the networks on "big data". In fact, authors did not train their networks on "big data", the statement is a misleading use of a buzzword.

Currently, we have trained the model on about 200 000 cells per epoch (as a comparison, the Cellpose dataset only contains 70 000 objects) and since we use augmented data (some of which is randomly generated), we more or less always have different cells presented to the model at each epoch. So altogether the model sees, as a rough estimation, up to 50 million (+/- 10 million) different cells during the entire training; this, to some extent, might be considered as big data. However, we agree with the reviewer that 'big data' is an ill-defined term and thereby we removed "big data" from the text.

- authors make a confusing statement about how manual correction of watershed result SHOULD improve network training. I am not sure what they mean and if it is an important point, quantitative evidence must be presented.

Reducing user input will inevitably reduce human bias and this could theoretically improve the learning, but on the other hand one might also argue that the model will learn the biases of the

© 2020. Published by The Company of Biologists under the terms of the Creative Commons Attribution License (https://creativecommons.org/licenses/by/4.0/).

watershed. As testing this hypothesis formally sounds extremely difficult (especially if that means segmenting all the training images entirely manually. Such task would easily take several months and up to a year). For this reason we removed the entire sentence from the text.

- "decently trained network" is not a appropriate expression

We replaced "to get a decently trained network" with “to train a network”

- "we flawlessly trained and run ... " is not a scientific statement.

We replaced "we flawlessly trained and ran ... " with “. We also successfully trained and ran …”

- the image quality of Figure 1 is low. Also, its message is trivial and should be expanded to highlight the strength of the contribution, i.e. user friendly software and GoogleCollab.

In the revised manuscript, we have modified the **Figure 1** to highlight the internal organization of EPySeg. Also, we now provide new tutorials reviewer 2 suggested that we think address user friendliness better than a figure.

- Figure 2 should be significantly expanded, showing more results on representative datasets.

As described previously, we now include more test samples in the figures and in the supplement of our manuscript.

- it is not clear what offset is referred to in the inset of the panel A. I don't see any Cellpose outlines.

We made an inset in **sup. Fig. 1E** that better shows the difference (this difference is also captured in the SEG scores found in **Table 1**). Of note, the Cellpose outline is significantly shifted away from the membrane maximum intensity (Cellpose detects the cell cytoplasm rather than the cell outline).

Reviewer 3 Advance Summary and Potential Significance to Field:

I have difficulties identifying advances made in this paper.

We here present a tool to segment epithelial tissues using deep learning, this tool may also be used more generally to train deep learning networks even by people with very limited experience with computers or images and without any equipment except an internet connection; we believe this tool does constitute an advance in the field.

Reviewer 3 Comments for the Author:

The submitted manuscript tackles a very important analysis step in many research projects. The presented work is in my view, unfortunately, not yet ready for publication.

Main points of concern:

- The quantification of obtained results is insufficient to understand where strength and weaknesses of the presented method lie.

In agreement with reviewer 3 comments, we have changed our quantification and now rely on the standard quantification methods used in the field **(SEG and AP scores, see methods)**. The results are qualitatively unchanged but the scores are much higher. This makes sense given that the new quantifications are less stringent than the ones we used previously. Altogether, we hope this fully addresses reviewer 3 concerns.

- The contribution by the authors is to make an existing network and training available via an open

© 2020. Published by The Company of Biologists under the terms of the Creative Commons Attribution License (https://creativecommons.org/licenses/by/4.0/).

source software package. No new technical tricks are introduced and even the combination of modules that are hooked up is very default and bears zero novelty.

Most existing models to segment cells and nuclei rely on the classical Unet architecture (with a classical Unet encoder and decoder) as reviewer#3 describes later. Here we used a different encoder (vgg16) and a Linknet architecture, a combination that was never used by any of the other tools mentioned (Cellpose, DenoiSeg, StarDist).

Regarding novelty, it's largely a matter of semantic. In Cellpose, for instance, the only modification to the classical Unet lies in the number of outputs (3 vs 1) and their type. Indeed, Cellpose produces 3 outputs, a horizontal and a vertical gradient along with a binary mask for the presence/absence of cells; these 3 outputs are then post-processed to produce the final segmentation mask. In our case, our model produces seven outputs, 5 of which are similar to watershed masks, while the remaining two can be seen as watershed seeds. Combining and post-processing these seven outputs produces a single segmentation outline for the cells (as shown in the revised **Figure 1**). To our knowledge this concept has not been used elsewhere.

Of note, by construction, Cellpose cannot segment non convex/complex cells, and Stardist is optimal for round shapes but less so for complex shapes ([1,2]), whereas our tool can handle complex shapes well **(see Fig. 2B, sup. Fig. 2B, B' and Table 1)**. In addition, Cellpose has a mandatory size parameter that dramatically impairs segmentation if not properly set (not shown) and prevents Cellpose from properly segmenting tissues where big and small cells co-exist (in such case Cellpose will only segment the most frequent of the two cell populations and ignore the other one **(sup. Fig. 1D)**, in contrast our tool only offers size filtering as an option, thereby allowing to segment tissues with a wide range of cell areas.

- Results are only compared to results obtained by Cellpose, and I expect that the pre-trained Cellpose model from Janelia Farms was used (the manuscript did not mention to have trained Cellpose for the specific data). This is bad because the comparison is flawed. While EPySeg is allowed to train on data very similar to the validation data, Cellpose must make the best out of the very different data it was trained on. Since Cellpose is also open source, nothing stops the authors from training on the same body of data and then do a proper comparison.

We have now trained Cellpose with the dataset we used to train EPySeg, but we got very poor results by doing so (see **Figure below**): cell outlines produced upon retraining of Cellpose with our dataset become extremely spiky, and less cells are detected with the retrained Cellpose compared to default/original pre- trained Cellpose model (see **Figure below**). These results, however, are not easy to interpret. Since we didn't train Cellpose with our augmented dataset, as Cellpose comes with its own data augmentation algorithms, the difference may come from there. Alternatively, the training of Cellpose, made on a very broad sample dataset (ranging from sea shells to cells), may make it more globally robust at segmenting any cellular object than ours, if that is the case, it is likely that training EPySeg with the Cellpose dataset may render it even more efficient than it actually is. However, we could not test the latter hypothesis as the training sample for Cellpose is not public and cannot be made available, for legal reasons, because images used to train Cellpose are gathered from the internet and contain numerous copyrighted material; already some images on the Cellpose webpage are copyrighted and exhibit a clear watermark* (e.g., see top left of the Cellpose image test image http://www.cellpose.org/static/images/img22.png, original copyrighted image https://www.pinterest.fr/pin/517139969706606279/?nic_v2=1a6WT9ICb).
*https://en.wikipedia.org/wiki/Digital_watermarking

© 2020. Published by The Company of Biologists under the terms of the Creative Commons Attribution License (https://creativecommons.org/licenses/by/4.0/).

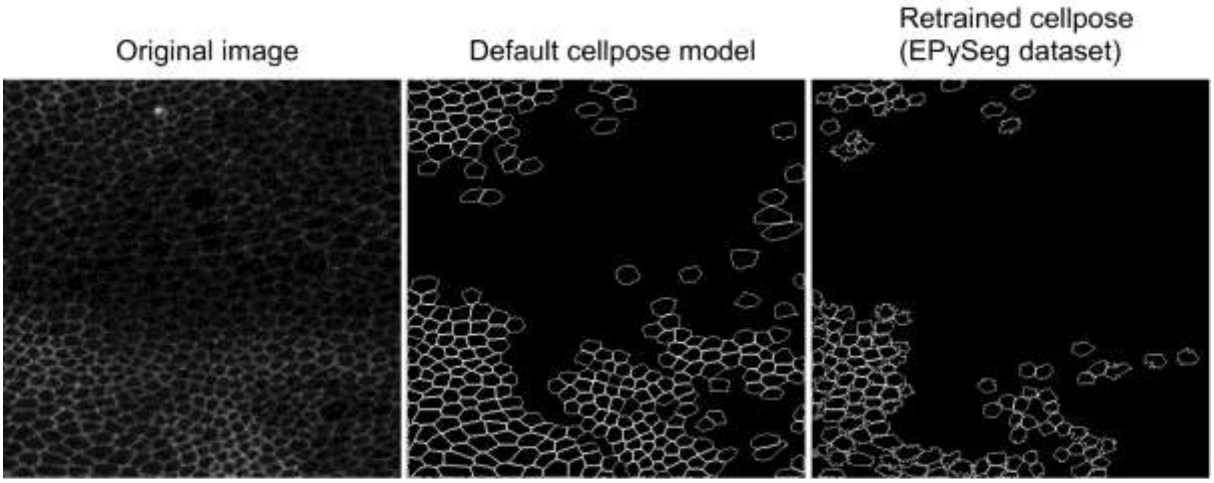

- Cellpose is not the only baseline that would be interesting to compare to. In fact, Cellpose is not even the most important baseline method. Obvious baselines would e.g. be a 3-Class U-Net (very basic and known to everyone), StarDist (Weigert et. Al.), or DenoiSeg (Buchholz, Prakash, et al.).

The reasons why we don't include StarDist in our comparison are many. First, StarDist has historically been developed to segment nuclei (and not cells), second StarDist cannot readily be used to segment epithelia like Cellpose and EPySeg do. And finally, Cellpose does compare itself to StarDist in their manuscript and concludes that it outperforms it at segmenting cells (and nuclei).

We did not see DenoiSeg straight away when it appeared on Arxiv and only got to know about it when it was announced on the ImageJ/FIJI forum. However, from our understanding, DenoiSeg is mainly of use when the size of the segmented training dataset is very small (below 38, for the n20 dataset, images according to the following link https://github.com/juglab/DenoiSeg/wiki/Quantitative-segmentation- comparison-for-Flywing-dataset). In any case, DenoiSeg also does require a big dataset for the denoising part of the model but the latter is rather easy to obtain (as it simply consists of original images with noise added to them). For large segmented datasets, a classical Unet-3 model without any parallel denoising is clearly as good as DenoiSeg (see 'Baseline' in the previous link). Since our training datasets and most epithelial datasets are much larger than the 38 images limit, the benefits of DenoiSeg for epithelial segmentation appear limited to both new and very divergent epithelia that would be poorly segmented by other existing tools such as Cellpose and EPySeg. Also, looking at the DenoiSeg manuscript, the segmentation accuracy (SEG score, [3]) for the wing epithelium is around 0.75 and this with or without the denoising module (see n20 in https://github.com/juglab/DenoiSeg/wiki/Quantitative-segmentation- comparison-for-Flywing-dataset) which is not that high. To further test Denoiseg, we ran the Denoiseg n20 (noise 20) fly wing code on the DenoiSeg fly wing dataset using 5 segmented images (5GT; i.e. we simply ran the python code for fly wings provided by the DenoiSeg authors), we then ran Cellpose (with the auto- optimal cell size parameter) and EPySeg segmentation (with its default parameters) on the DenoiSeg n20 test set (see **Figure below**) to be able to directly compare the three tools. Importantly, Cellpose and EpySeg were not re-trained on the dataset. We then computed the average SEG and AP scores for the 42 test images of DenoiSeg n20 trained with 5 ground truth masks (5GT) (with a stringent IoU of 0.7 for computing the AP score) for the three tools. The SEG score was as follows EPySeg=0,838 > Cellpose= 0,746 > DenoiSeg=0,512. For the AP score, we obtained EpySeg=0,966 > Cellpose=0,924 > DenoiSeg= 0,467. Importantly, we could not reach the values described in the DenoiSeg manuscript (AP=0.882 and SEG= 0.724), values were lower in our hands and scores varied highly from run to run when the Denoiseg model was trained with only 5 GT. We then ran Denoiseg with 76 GT masks, in order to be in optimal conditions, and computed the average score for the 42 denoiseg test images. This time we obtained the published scores for Denoiseg: AP=0.952 SEG=0.755; we note that Denoiseg now outperforms Cellpose but is below EPySeg both for AP and SEG scores (very largely for the latter).

We have removed unpublished data provided for the referees in confidence.

© 2020. Published by The Company of Biologists under the terms of the Creative Commons Attribution License (https://creativecommons.org/licenses/by/4.0/).

Finally, we ran the GT76 (optimal) trained Denoiseg model on other epithelial samples, including fly wing epithelia. In all cases, we obtained rather poor segmentation with Denoiseg **(see Figure below)**, suggesting that Denoiseg is a terribly efficient, yet very specialized model as compared to EPySeg and Cellpose. That is why we chose not to include Denoiseg in our comparison.
Also to clarify why we only compare EPySeg to Cellpose, we added the following sentence to the main text 'We compared our software to Cellpose, the only software available to date that can segment cells without the need for prior model training [1].'

We have removed unpublished data provided for the referees in confidence.

- The authors suggest to use the trained EpySeg model as a "generalist neural network" (page 1). While in the context of segmentation this can indeed work ok, it is clear that this comes with strong limitations. New data that is different to the training data will not work well at all and this is also the reason why the comparison with Cellpose is so deeply flawed.

The truncated sentence the reviewer refers to makes a clear mention that the tool is to be used on 'epithelial tissues'. However, for further clarification we removed the term 'generalist' from the sentence. We now show that our model performs well on data significantly different from the one used for training the model, such as plant samples with irregular cells and vertebrate heart cells stained with actin. We hope this reassures the reviewer on the robustness of our training.

Also, regarding the fairness of the comparison with Cellpose, please note that we don't use the default Cellpose parameters (that would perform much less well than what we show) but let it calculate the optimal cell size so that it can detect cells in optimal conditions. We also show that when specifically trained on our dataset Cellpose does not perform better, suggesting Cellpose training may already be optimal and it may be hard for it to better segment epithelial samples than it already does. Also, we clearly state that we compare the tool with respect to epithelia and mention in the **Table** that on divergent samples (such as cells in culture) Cellpose performs better. Furthermore, we added a sentence in the main text stating that EPySeg 'is likely to be less efficient at segmenting non-cellular objects than Cellpose since it was not trained to accomplish such tasks.', so altogether we think we are doing a fair comparison of the two tools.

- The description of augmentation and training is insufficient. Whats the learning rate? Does it adapt during training? What was the optimizer, what's the precise network architecture? How can IoU be the loss (IoU is non-differentiable, see also https://stackoverflow.com/questions/40475246/why-does-one-not-use- iou-for-training).

We used 'Adam' as the optimizer.

We rewrote the 'Convolutional neural network building and training' method section, it now includes the optimizer, batch size and learning rate details. Our previous description was broad because the tool included several models, we are now more specific since we only include a single model in our tool.

Our model has a Vgg16-Linknet architecture, the details are listed below. Of note, the architecture is displayed in the log window of the software (see a copy of the log below), so we don't think it is useful to add this to the manuscript (also because it takes a lot of space and will not be of much use to the reader). Finally, to help the reader, we added the following sentence in the methods section (Convolutional neural network building and training): 'Of note, the detailed model architecture is shown in the log window of the software upon loading.'

#Model architecture:

Layer (type)	Output Shape	Param #	Connected to
input_1 (InputLayer)	[(None, None, None, 1)]	0	

© 2020. Published by The Company of Biologists under the terms of the Creative Commons Attribution License (https://creativecommons.org/licenses/by/4.0/).

block1_conv1 (Conv2D)	(None, None, None, 64)	640	input_1[0][0]
block1_conv2 (Conv2D)	(None, None, None, 64)	36928	block1_conv1[0][0]
block1_pool (MaxPooling2D)	(None, None, None, 64)	0	block1_conv2[0][0]
block2_conv1 (Conv2D)	(None, None, None, 128)	73856	block1_pool[0][0]
block2_conv2 (Conv2D)	(None, None, None, 128)	147584	block2_conv1[0][0]
block2_pool (MaxPooling2D)	(None, None, None, 128)	0	block2_conv2[0][0]
block3_conv1 (Conv2D)	(None, None, None, 256)	295168	block2_pool[0][0]
block3_conv2 (Conv2D)	(None, None, None, 256)	590080	block3_conv1[0][0]
block3_conv3 (Conv2D)	(None, None, None, 256)	590080	block3_conv2[0][0]
block3_pool (MaxPooling2D)	(None, None, None, 256)	0	block3_conv3[0][0]
block4_conv1 (Conv2D)	(None, None, None, 512)	1180160	block3_pool[0][0]
block4_conv2 (Conv2D)	(None, None, None, 512)	2359808	block4_conv1[0][0]
block4_conv3 (Conv2D)	(None, None, None, 512)	2359808	block4_conv2[0][0]
block4_pool (MaxPooling2D)	(None, None, None, 512)	0	block4_conv3[0][0]
block5_conv1 (Conv2D)	(None, None, None, 512)	2359808	block4_pool[0][0]
block5_conv2 (Conv2D)	(None, None, None, 512)	2359808	block5_conv1[0][0]
block5_conv3 (Conv2D)	(None, None, None, 512)	2359808	block5_conv2[0][0]
block5_pool (MaxPooling2D)	(None, None, None, 512)	0	block5_conv3[0][0]
center_block1_conv (Conv2D)	(None, None, None, 512)	2359296	block5_pool[0][0]
center_block1_bn (BatchNormalization)	(None, None, None, 512)	2048	center_block1_conv[0][0]
center_block1_relu (Activation)	(None, None, None, 512)	0	center_block1_bn[0][0]
center_block2_conv (Conv2D)	(None, None, None, 512)	2359296	center_block1_relu[0][0]

© 2020. Published by The Company of Biologists under the terms of the Creative Commons Attribution License (https://creativecommons.org/licenses/by/4.0/).

center_block2_bn (BatchNormalization)	(None, None, None, 512)	2048	center_block2_conv[0][0]
center_block2_relu (Activation)	(None, None, None, 512)	0	center_block2_bn[0][0]
decoder_stage0a_conv (Conv2D)	(None, None, None, 128)	65536	center_block2_relu[0][0]
decoder_stage0a_bn (BatchNormalization)	(None, None, None, 128)	512	decoder_stage0a_conv[0][0]
decoder_stage0a_relu (Activation)	(None, None, None, 128)	0	decoder_stage0a_bn[0][0]
decoder_stage0_upsamplin [illegible]	(None, None, [illegible]	0	decoder_stage0a_relu[0][0]
decoder_stage0b_conv (Conv2D)	(None, None, None, 128)	147456	decoder_stage0_upsampling[0][0]
decoder_stage0b_bn (BatchNormalization)	(None, None, None, 128)	512	decoder_stage0b_conv[0][0]
decoder_stage0b_relu (Activation)	(None, None, None, 128)	0	decoder_stage0b_bn[0][0]
decoder_stage0c_conv (Conv2D)	(None, None, None, 512)	65536	decoder_stage0b_relu[0][0]
decoder_stage0c_bn (BatchNormalization)	(None, None, None, 512)	2048	decoder_stage0c_conv[0][0]
decoder_stage0c_relu (Activation)	(None, None, None, 512)	0	decoder_stage0c_bn[0][0]
decoder_stage0_add (Add)	(None, None, None, 512)	0	decoder_stage0c_relu[0][0]<br>block5_conv3[0][0]
decoder_stage1a_conv (Conv2D)	(None, None, None, 128)	65536	decoder_stage0_add[0][0]
decoder_stage1a_bn (BatchNormalization)	(None, None, None, 128)	512	decoder_stage1a_conv[0][0]
decoder_stage1a_relu (Activation)	(None, None, None, 128)	0	decoder_stage1a_bn[0][0]
decoder_stage1_upsampling (UpSampling2D)	(None, None, None, 128)	0	decoder_stage1a_relu[0][0]
decoder_stage1b_conv (Conv2D)	(None, None, None, 128)	147456	decoder_stage1_upsampling[0][0]
decoder_stage1b_bn (BatchNormalization)	(None, None, None, 128)	512	decoder_stage1b_conv[0][0]
decoder_stage1b_relu (Activation)	(None, None, None, 128)	0	decoder_stage1b_bn[0][0]
decoder_stage1c_conv (Conv2D)	(None, None, None, 512)	65536	decoder_stage1b_relu[0][0]

© 2020. Published by The Company of Biologists under the terms of the Creative Commons Attribution License (https://creativecommons.org/licenses/by/4.0/).

decoder_stage1c_bn (BatchNormalization)	(None, None, None, 512)	2048	decoder_stage1c_conv[0][0]
decoder_stage1c_relu (Activation)	(None, None, None, 512)	0	decoder_stage1c_bn[0][0]
decoder_stage1_add (Add)	(None, None, None, 512)	0	decoder_stage1c_relu[0][0]<br>block4_conv3[0][0]
decoder_stage2a_conv (Conv2D)	(None, None, None, 128)	65536	decoder_stage1_add[0][0]
decoder_stage2a_bn (BatchNormalization)	(None, None, None, 128)	512	decoder_stage2a_conv[0][0]
decoder_stage2a_relu (Activation)	(None, None, None, 128)	0	decoder_stage2a_bn[0][0]
decoder_stage2_upsamplin g (UpSampling2D)	(None, None, None, 128)	0	decoder_stage2a_relu[0][0]
decoder_stage2b_conv (Conv2D)	(None, None, None, 128)	147456	decoder_stage2_upsampling[0][0]
decoder_stage2b_bn (BatchNormalization)	(None, None, None, 128)	512	decoder_stage2b_conv[0][0]
decoder_stage2b_relu (Activation)	(None, None, None, 128)	0	decoder_stage2b_bn[0][0]
decoder_stage2c_conv (Conv2D)	(None, None, None, 256)	32768	decoder_stage2b_relu[0][0]
decoder_stage2c_bn (BatchNormalization)	(None, None, None, 256)	1024	decoder_stage2c_conv[0][0]
decoder_stage2c_relu (Activation)	(None, None, None, 256)	0	decoder_stage2c_bn[0][0]
decoder_stage2_add (Add)	(None, None, None, 256)	0	decoder_stage2c_relu[0][0]<br>block3_conv3[0][0]
decoder_stage3a_conv (Conv2D)	(None, None, None, 64)	16384	decoder_stage2_add[0][0]
decoder_stage3a_bn (BatchNormalization)	(None, None, None, 64)	256	decoder_stage3a_conv[0][0]
decoder_stage3a_relu (Activation)	(None, None, None, 64)	0	decoder_stage3a_bn[0][0]
decoder_stage3_upsamplin g (UpSampling2D)	(None, None, None, 64)	0	decoder_stage3a_relu[0][0]
decoder_stage3b_conv (Conv2D)	(None, None, None, 64)	36864	decoder_stage3_upsampling[0][0]
decoder_stage3b_bn (BatchNormalization)	(None, None, None, 64)	256	decoder_stage3b_conv[0][0]
decoder_stage3b_relu (Activation)	(None, None, None, 64)	0	decoder_stage3b_bn[0][0]

© 2020. Published by The Company of Biologists under the terms of the Creative Commons Attribution License (https://creativecommons.org/licenses/by/4.0/).

decoder_stage3c_conv (Conv2D)	(None, None, None, 128)	8192	decoder_stage3b_relu[0][0]
decoder_stage3c_bn (BatchNormalization)	(None, None, None, 128)	512	decoder_stage3c_conv[0][0]
decoder_stage3c_relu (Activation)	(None, None, None, 128)	0	decoder_stage3c_bn[0][0]
decoder_stage3_add (Add)	(None, None, None, 128)	0	decoder_stage3c_relu[0][0]<br>block2_conv2[0][0]
decoder_stage4a_conv (Conv2D)	(None, None, None, 32)	4096	decoder_stage3_add[0][0]
decoder_stage4a_bn (BatchNormalization)	(None, None, None, 32)	128	decoder_stage4a_conv[0][0]
decoder_stage4a_relu (Activation)	(None, None, None, 32)	0	decoder_stage4a_bn[0][0]
decoder_stage4_upsampling (UpSampling2D)	(None, None, None, 32)	0	decoder_stage4a_relu[0][0]
decoder_stage4b_conv (Conv2D)	(None, None, None, 32)	9216	decoder_stage4_upsampling[0][0]
decoder_stage4b_bn (BatchNormalization)	(None, None, None, 32)	128	decoder_stage4b_conv[0][0]
decoder_stage4b_relu (Activation)	(None, None, None, 32)	0	decoder_stage4b_bn[0][0]
decoder_stage4c_conv (Conv2D)	(None, None, None, 16)	512	decoder_stage4b_relu[0][0]
decoder_stage4c_bn (BatchNormalization)	(None, None, None, 16)	64	decoder_stage4c_conv[0][0]
decoder_stage4c_relu (Activation)	(None, None, None, 16)	0	decoder_stage4c_bn[0][0]
conv2d (Conv2D)	(None, None, None, 7)	1015	decoder_stage4c_relu[0][0]
sigmoid (Activation)	(None, None, None, 7)	0	conv2d[0][0]

Regarding the comment of the reviewer on the use of IoU as a loss, we would argue that the first answer in the Stackoverflow post already refers to an Arxiv paper using an IoU loss; in that case it is applied to bounding boxes. In our case, a more suited reference, could be, for example, the article by Rahman and Wang (https://www.cs.umanitoba.ca/~ywang/papers/isvc16.pdf) that describes an IoU loss applied to binary images and that shows how it outperforms the accuracy loss and optimizes semantic segmentation. There are also several implementations of IoU as a loss throughout the internet (e.g. https://github.com/qubvel/segmentation_models/blob/master/segmentation_models/losses.py, https://www.kaggle.com/c/data-science-bowl-2018/discussion/51553, …).

© 2020. Published by The Company of Biologists under the terms of the Creative Commons Attribution License (https://creativecommons.org/licenses/by/4.0/).

All data is presented at each epoch, but does that mean all pixels are presented (since small tiles are fed)?

Yes, pretty much all the data and all the pixels/cells are presented at each epoch because the images are fully split into tiles dynamically. However, in order to keep the total number of tiles/batches constant, we ensure that the images always have the same size after augmentation. In some cases, such as for the Zoom/magnification augmentation, images must be trimmed to the original image size and this inevitably causes some cell loss on the way. In contrast, some data augmentation, such as flip, do not change image size and then all cells are passed. Importantly, the full dataset is not stored in the memory as it is for some competing tools, this means the model can virtually be trained on an infinite number of datasets of any size.

Are the same augmented versions shown over and over, or is the augmentation different between epochs? Which dataset was using which patch and batch size? Etc. etc.

No, the augmentation is not static (generated once and used throughout training) as done for example for StarDist training, it is dynamically generated at every step and randomly performed within a given range (random rotation, x-y translation, flip, noise, …). Please *see the* 'Convolutional neural network building and training' section of the material and methods for patch and batch size. We note, however, that the latter two parameters were not of critical importance in our tests, whereas data augmentation was (not all augmentations have a positive impact on final segmentation, but we haven't conducted a detailed study of this).

- The 'segmentation quality' metric introduced is strange and it is really not needed to introduce such a metric (there is already too many around that even make sense).

This comment is somehow overlapping with the next one, so we'll answer later.

- The metric is: (#correct - #overseg - #underseg)/#truecells. Now, each over or undersegmentation removes 1 from #correct, but causes another subtraction by 1 in the nominator. Hence, this metric will be 0 as soon as half the cells are over or undersegmentations. Weird! I suggest for example to use AP scores or any other established metric.

We thank the reviewer for pointing to us the irrationality of our metric. In the revised manuscript, we now stick to the standard quantifications (SEG and AP scores) used in the field as mentioned previously.

Other concerns:

- All work only applies to 2D data and this might go into the title to not elicit false hopes.
Given that 3D images are merely series of 2D images along the Z axis, nothing technically prevents the user from obtaining a 3D segmentation by splitting it into a series of 2D images (that would also be much less demanding memory-wise than using directly a 3D model). Also, we leave open the possibility to include 3D unets or alike in the software in the near future; so altogether, we prefer to remain open in the main text. Finally, 2D is specifically mentioned, in bold, in the first line of the software description page (https://github.com/baigouy/EPySeg), so we believe there is sufficient information for anybody willing to download the software to decide whether or not it can be useful for him/her and we believe no further action is required.

- Several claims in the paper are not right or overly emphasized.

- Page 1: "Training cannot be done directly in FIJI/ImageJ" - yes, it can! DenoiSeg comes with such an option (https://imagej.net/DenoiSeg).

The author is right (now), DenoiSeg is 'finally' an example of a training directly done in FIJI, we added a reference to DenoiSeg in the main text. Of course, training in FIJI is still not that easy as it requires the system and FIJI to be properly set and to have a compatible graphic card, to rely on an

© 2020. Published by The Company of Biologists under the terms of the Creative Commons Attribution License (https://creativecommons.org/licenses/by/4.0/).

aging tensorflow driver that may never be updated. Also, we note that the FIJI DenoiSeg module does not output a segmentation mask directly, as one would expect, but simply the raw model output (consisting of 4 channels) and regarding the python code provided with Denoiseg, we still had to derive our own code to be able to easily get the DenoiSeg segmentation mask out of the trained model. So altogether, this would still prevent a significant number of users from easily using this tool. See also our detailed answer below regarding the ease of use of deep learning.

- Page 1: I'm not sure if it is fair to say that the computer vision field got "revamped" at certainly the need for user correction is by far not "alleviated". Deep learning improved things, but segmentation remains a hard problem that is by far not automatically solvable.

We have changed the text to: 'Over the past few years, deep learning, and more particularly convolutional neural networks (CNNs), has reshaped the computer vision field. In particular, deep learning approaches should be beneficial for image segmentation as they could, in theory, reduce or even eliminate the need for end-user correction of the segmentation output.'

- "the majority of scientific computers are not deep learning-ready", depending on what the authors mean, I might have to strongly disagree. Today it is hard to even buy a laptop that is not powerful enough for a number of useful deep learning (please exclude Mac computers here, but for very different reasons). Anyway, every single microscope workstation should be more then enough for EPySeg, Cellpose, et al.

Deep learning is largely restricted to computers equipped with a recent NVIDIA graphic cards, which represents only a subset of the desktop and an even smaller portion of the laptop computers, in addition most professional working stations are by default equipped with deep learning incompatible/poorly suited graphic cards (e.g. cards from the NVIDIA Quadro family). Also, as mentioned, current and future Mac computers do and will not support deep learning training for tensorflow models through graphic cards (https://www.tensorflow.org/install). This is a big problem given the predominance of Apple computers among biologists. So altogether, we are indeed just left with a few recent workstations where the graphic card(s) were wisely chosen to use deep learning, this is in our mind represents a serious limitation for the end-user (especially given that training may require up to several days). So altogether, we believe our alternative solution is highly valuable especially when it comes to training CNNs.

- I'm not sure about the validity of the hypothesis that EPySeg training is working better due to the non- human (but Watershed) origin of the training data. I would like to see such a claim backed up by adequate control experiments. It would, for example, be very interesting to see if a network trained on such data has the tendency to put the outline of cells at roughly constant intensity values (such as Watershedding does). Anyways, at all the crucial places the network is again trained on user annotations (curations) and the argument seems not to hold any longer.

Removing human input will remove human bias that may impair learning; this is why we added this sentence. However, the reviewer is right and there is unfortunately no easy way to test this statement (since it's virtually impossible to re-segment manually all of our training datasets), so we removed the sentence.

- Authors say that EPySeg does not work on cells in culture. This is of course true, because it was not trained for this use-case. Still, could a version of EPySeg exist that was trained on this data? If so, why not do it and serve a much larger community. If not - why not?

Indeed, our tool is specifically designed and optimized to segment epithelial cells so we haven't tried to train a network to segment cells in culture nor nuclei. In addition, we think that given the difficulty of training a model, even besides having the equipment for it, it is of great interest for the community to have a series of pre-trained specialized and optimized models to accomplish specific segmentation tasks, especially when this segmentation is highly time-consuming, which is the case for epithelial segmentation.

- The caption of Figure 2 does not write their own method name correctly...

© 2020. Published by The Company of Biologists under the terms of the Creative Commons Attribution License (https://creativecommons.org/licenses/by/4.0/).

Indeed, we thank the reviewer for spotting this typo, we have changed the text.

- A table to compare results would be important. The supplementary table comes without caption and must be enriched by other baselines to make sense.

We have written a caption for the **Table**

- One would wish for many more qualitative examples of inputs and results in the supplement.

We have now added new data to the main text and supplement, we hope this addresses the reviewer comment.

References

1 Stringer, C., Wang, T., Michaelos, M. & Pachitariu, M. Cellpose: a generalist algorithm for cellular segmentation. *bioRxiv* (2020).

2 Schmidt, U., Weigert, M., Broaddus, C. & Myers, G. Cell Detection with Star-convex Polygons. *arXiv:1806.03535 [cs]* **11071**, 265-273, doi:10.1007/978-3-030-00934-2_30 (2018).

3 Ulman, V. *et al.* An objective comparison of cell-tracking algorithms. *Nature Methods* **14**, 1141- 1152, doi:10.1038/nmeth.4473 (2017).

<u>Second decision letter</u>

MS ID#: DEVELOP/2020/194589

MS TITLE: EPySeg: a coding-free solution for automated segmentation of epithelia using deep learning

AUTHORS: Benoit Aigouy, Claudio Cortes, Shanda Liu, and Benjamin Prud'homme
ARTICLE TYPE: Techniques and Resources Report

I am happy to tell you that your manuscript has been accepted for publication in Development, pending our standard ethics checks.

<u>Reviewer 1</u>

Advance summary and potential significance to field

This study provides a useful tool that should encourage more researchers to try AI approaches for data analysis, therefore I am happy to support publication in Development.

Comments for the author

The authors have made changes that addressed all my questions.

<u>Reviewer 2</u>

Advance summary and potential significance to field

Aigouy et al. present a good revision of the initial submission. They present now a very concise presentation of the EpySeq tool aimed at the biology users and presented to the appropriate research community of Development. My requested revisions have been largely implemented.

© 2020. Published by The Company of Biologists under the terms of the Creative Commons Attribution License (https://creativecommons.org/licenses/by/4.0/).

Comments for the author

We remain divided on whether this represents a significant advance in the field of computer vision. I do not agree with the authors dismissal of StarDist and CellPose as "using basic Unet models with varying number of model and output post-processing". Try to submit EpySeq to a computer vision conference and see what happens. Nevertheless this is a pointless academic dispute that has no bearing on the general usefulness of the EpySeq software for biologists. This is a nice and performant tool and I am sure it has a bright future.

Reviewer 3

Advance summary and potential significance to field

The authors improved the manuscript to some degree.
Unfortunately, this reviewer does not have the impression that the manuscript was changed sufficiently and feels that in many cases a path of lowest resistance was taken instead of striving for the best possible paper to submit.

Comments for the author

1. The main criterion for publication if a Research Article or Report in Development is that a paper should make a significant and novel contribution to our understanding of developmental mechanisms. Studies lacking such a contribution, no matter how meticulous, are not acceptable for publication.

From a computational point of view the novelty is marginal and not at all described sufficiently (or well).
If the editor decides that the existence of a tool for epithelia segmentation is novel enough, I will respect this decision.

2. Development has a 'Techniques and Resources' section. Articles submitted to this section should be assessed according to the novelty and importance for the community of the technique or resource reported.

See my comment to (1). Maybe the presented work can be seen as a breakthrough resource in the field of epithelia 2D segmentation, but I am not the right person to judge and it would draw a dim picture about the state of this field.

3. Development is only able to accept around 30% of the papers it receives. Editorial decisions are reached based on input from two or more usually three referees. Should the editor receive conflicting reports, he/she may contact you for further advice after your report has been submitted.

Unless the manuscript changes significantly in accordance to the very valuable feedback given by all 3 reviewers of round 1, I would appreciate to not have to spend more time reading endless comments and justifications by the authors (which, unfortunately, often miss the point of the original comment).

4. Development operates a 'cross-referee commenting' system, giving reviewers the option to view and comment on each others' reports before the editor makes a decision on the paper. Once all reports have been returned, you will receive an email inviting you to provide further feedback. We appreciate your participation in this process, which we find very helpful in making well-informed decisions and clearer guidance to authors.

Happy to contribute there once available.

5. We expect reviewers to review papers in a respectful manner and not to write anything that could cause offense or be defamatory. Please take care to ensure that any statements are factually

© 2020. Published by The Company of Biologists under the terms of the Creative Commons Attribution License (https://creativecommons.org/licenses/by/4.0/).

supported, and that opinions stated are genuinely held and well-justified. On rare occasions where the editors of the journal are concerned that papers have not been reviewed according to these principles, we may contact the reviewer and request changes to the report before it is transmitted to the authors.

I hope there will be no reason to contact me to reword my review.
I, indeed, dislike the style of this paper quite profoundly, but I acknowledge that I am not a developmental biologist and might not see the urgency of such a tool existing.
Still, a new method should be explained well, and the technical part of this paper is still not written well or complete. I see this paper being stuck somewhere between targeting users and being simple, but then at the same time going deep enough to make me miss a thorough description of the method.

The most fundamental flaw, in my point of view, is the lack of better comparison to other existing methods.
The authors obviously did follow up on my random suggestions, but still do not compare to results obtained with StarDist. But other state-of-the-art methods I did not mention last time were not considered at all (e.g. PatchPerPix, MaskRCNN, etc.).

I am wondering to what degrees the authors are themselves to be considered experts in the field of deep-learning based instance segmentation. There are many places (not only in the manuscript, but also in the rebuttal) where wrong, misleading, or imprecise statements are made (Cellpose, for example, can easily segment non-convex objects. StarDist can easily segment the kind of cells that are 'encircled' by the membrane markers used in the manuscript, etc. etc.

© 2020. Published by The Company of Biologists under the terms of the Creative Commons Attribution License (https://creativecommons.org/licenses/by/4.0/).
